# Supplementary material for: A Form of Non-Volatile Solid-like Hexadecane Found in Micron-Scale Silica Microtubule
Source: Materials (Basel). 2022 Dec 20;16(1):9. doi: 10.3390/ma16010009 (PMC9820975; doi:10.3390/ma16010009)
Supplement: Supplementary file 1 [file materials-16-00009-s001.zip › materials-2037289-supplementary.pdf]

## S1: TEM samples

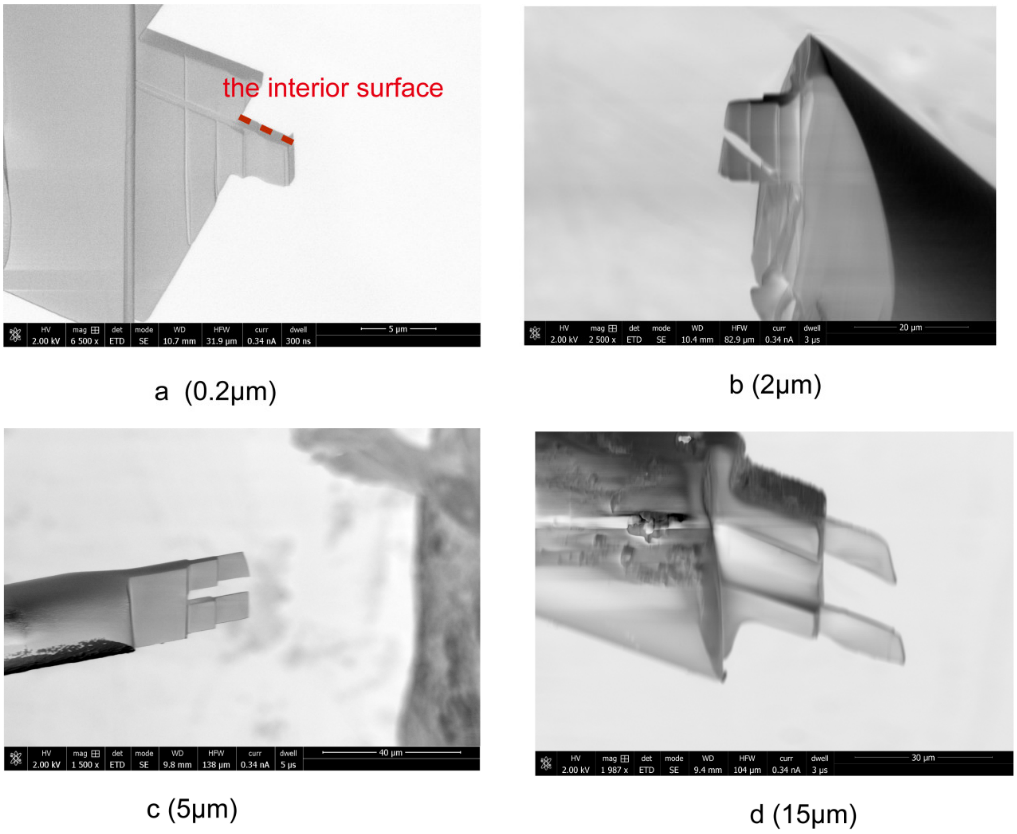


**Figure S1.** SEM images of TEM samples fabricated with microtubules with different diameters.

## S2: Energy Dispersive Spectroscopy Data of hexadecane on the inner surface of silica microtubules 5μm in ID.

Figure S2(b) shows that with the exception of the three peaks for the carbon, the silicon and the oxygen in the energy spectrum corresponding to the $SiO_2$ microtubule and the hexadecane that was at the wall of the microtubule, there was a very small amount of copper comes from the copper mesh and some gallium elements come from gallium ions to make slices by dual beam scanning electron microscope.

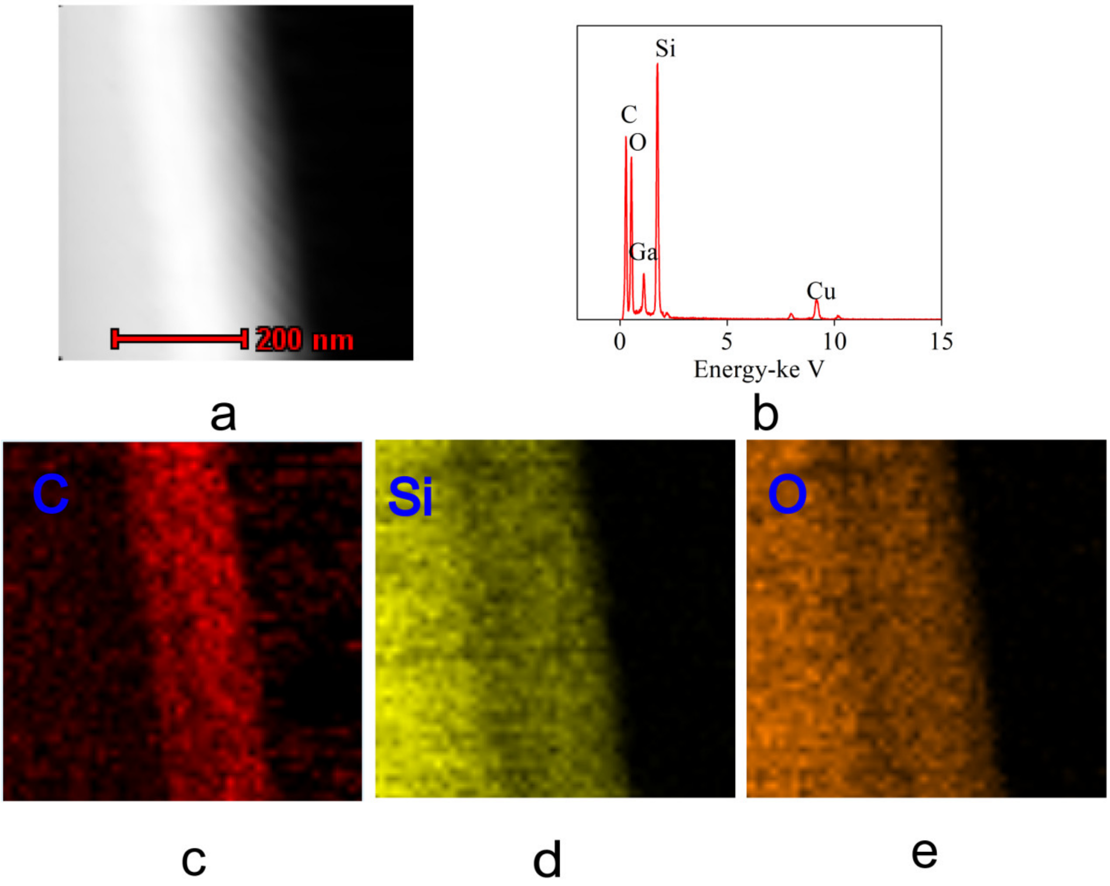


**Figure S2.** Energy Dispersive Spectroscopy Data of hexadecane on the inner surface of silica microtubules (inner diameter of 5μm). Figure S2(**a**) is the high-angle annular dark-field(HAADF) imaging of the inner wall of the microtubule after infiltration with hexadecane. Figure S2(**b**–**e**) are the energy spectrum data and the EDS areal scans of the C, Si, O elements taken of the same area as in Figure S2(a).

**S3: Energy Dispersive Spectroscopy Data of hexadecane slices in silica microtubules 0.4μm in ID.**

Figure S3(a) is the high-angle annular dark-field(HAADF) imaging of hexadecane slices. Figure S3(b) shows that carbon, the silicon, the oxygen .Figure S3(c–d) shows that carbon, the silicon, the oxygen in Figure S3(a).Figure S3 shows that there are only C elements in hexadecane slices by EDS

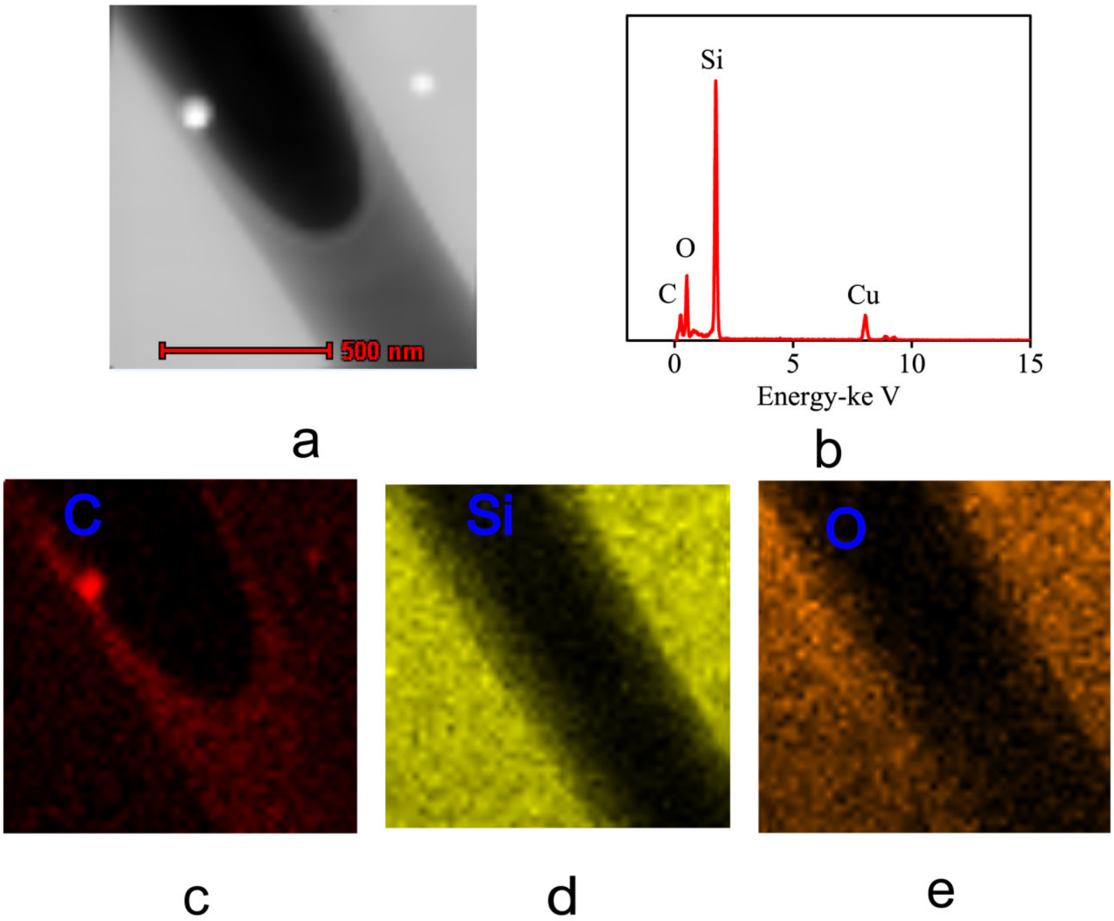


**Figure S3.** Energy Dispersive Spectroscopy Data of hexadecane in silica microtubules 0.4μm in ID. Figure S3(**a**) shows the high-angle annular dark-field (HAADF) imaging of hexadecane in microtubule 0.4 μ m in ID. Figure S3(**b**–**e**) are the energy spectrum data and the EDS areal scans of the C, Si and O elements taken of the same area as in Figure S3(a).

**S4: Energy Dispersive Spectroscopy Data of hexadecane on the inner surface of silica pores 0.3μm in ID.**

This hole has been in the air for 8 weeks since it was made. Soak in hexadecane for 1 minute before testing. Figure S4(a) is the high-angle annular dark-field (HAADF) imaging of hexadecane in submicron silica pores 0.3μm in ID. Figure S4(b) shows that carbon, the silicon, the oxygen .Figure S4 (c–d) shows that carbon, the silicon, the oxygen in Figure S4(a).Figure S4 shows that there are only C elements in hexadecane slices by EDS. It can be seen from the figure that only the hexadecane aggregates in the submicron pores.

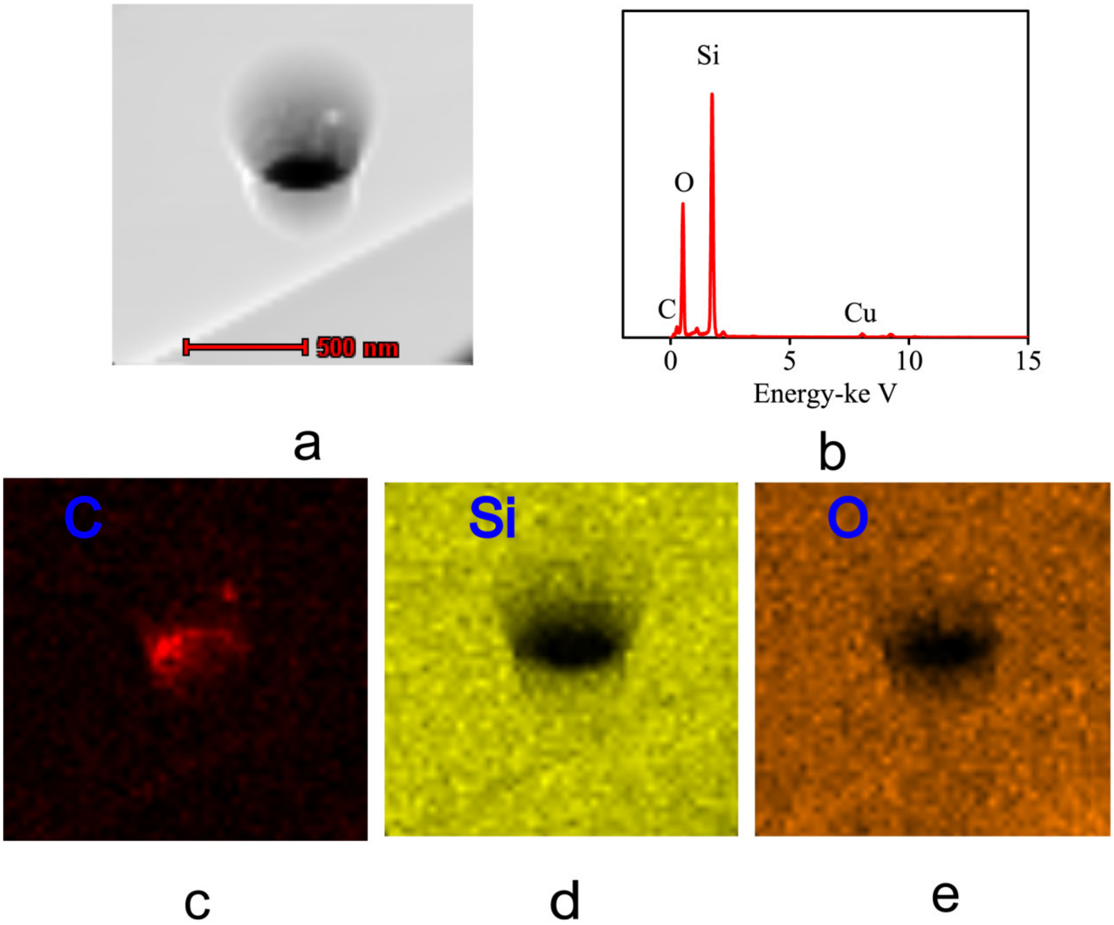


**Figure S4.** Energy Dispersive Spectroscopy Data of hexadecane in Submicron silica pores 0.3μm in ID. Figure S4(**a**) shows the high-angle annular dark-field (HAADF) imaging of hexadecane in microtubule 0.3μm in ID. Figure S4(**b**–**e**) are the energy spectrum data and the EDS areal scans of the C, Si and O elements taken of the same area as in Figure S4(a).
